# Towards a metabolomic approach to investigate iron–sulfur cluster biogenesis

**DOI:** 10.1002/iub.2618

**Published:** 2022-04-27

**Authors:** Mauro Marengo, Alex Fissore, Simonetta Oliaro‐Bosso, Salvatore Adinolfi, Annalisa Pastore

**Affiliations:** ^1^ Department of Drug Science and Technology University of Turin Turin Italy; ^2^ Department of Basic and Clinical Neuroscience The Maurice Wohl Institute, King's College London, Denmark Hill Campus London UK

**Keywords:** CyaY, desulfurase, enzymatic activity, frataxin, Friedreich's ataxia, iron–sulfur cluster biogenesis

## Abstract

Iron–sulfur clusters are prosthetic groups that are assembled on their acceptor proteins through a complex machine centered on a desulfurase enzyme and a transient scaffold protein. Studies to establish the mechanism of cluster formation have so far used either *in vitro* or *in vivo* methods, which have often resulted in contrasting or non‐comparable results. We suggest, here, an alternative approach to study the enzymatic reaction, that is based on the combination of genetically engineered bacterial strains depleted of specific components, and the detection of the enzymatic kinetics in cellular extracts through metabolomics. Our data prove that this *ex vivo* approach closely reproduces the *in vitro* results while retaining the full complexity of the system. We demonstrate that co‐presence of bacterial frataxin and iron is necessary to observe an inhibitory effect of the enzymatic activity of bacterial frataxin. Our approach provides a new powerful tool for the study of iron–sulfur cluster biogenesis.

**Abbreviations**
DMPD: N,N‐dimethyl‐p‐phenylenediamine
DTT: dithiothreitol
FRDA: Friedreich's ataxia
NTA: nitrilotriacetic acid
OD: optical density
PLP: pyridoxal 5'‐phosphate
PVDF: polyvinylidene difluoride
SDS: sodium dodecyl sulfate
UV: ultraviolet

## 1 INTRODUCTION

Frataxin, an essential protein highly conserved in most organisms, is involved in the neurodegenerative disease Friedreich's ataxia (FRDA) in humans.^1^, ^2^ This disease is caused by the expansion of a triplet in the first intron of the FRDA gene which leads to the gene partial silencing and consequently decreased levels of the gene product frataxin.^3^ More than 25 years after the identification of the FRDA gene,^3^ a large set of data have been gathered in many organisms (both eukaryotic and prokaryotic) which have established that frataxins are iron‐binding proteins and regulators of the iron–sulfur cluster biogenesis machine.^4^, ^5^, ^6^, ^7^, ^8^, ^9^ This is an important metabolic pathway that is finely tuned to enzymatically convert cysteine into alanine and a very active persulfide group by the desulfurases Nfs1 (eukaryotes) or IscS (prokaryotes). The persulfide is then transferred to the scaffold protein (Isu/IscU in eukaryotes/prokaryotes, respectively) where the cluster is transiently assembled.^10^, ^11^, ^12^, ^13^ Other proteins such as ferredoxin and two co‐chaperones assist the process.

While all the components of the machine are now known, it has been more difficult to understand the detailed mechanism by which frataxins function and their precise role in the pathway. *In vitro*, frataxins bind stoichiometrically the desulfurase and influence the speed of conversion of cysteine to alanine in a species‐related fashion^14^: eukaryotic frataxins have the effect of stabilizing the desulfurase Nfs1 fold and enhance the speed of the conversion reaction. On the other hand, the bacterial frataxin ortholog CyaY was shown to inhibit the IscS enzymatic activity likely by decreasing the rate of the detachment of IscU from IscS and/or impairing the movements of a flexible loop carrying out the catalytic cysteine from the active site to the scaffold protein acceptor.^15^ It was also noted that, *in vitro*, the bacterial desulfurase IscS is active also in the absence of CyaY,^5^, ^15^, ^16^ whereas eukaryotic Nfs1 is inactive in the absence of frataxin.^17^, ^18^, ^19^, ^20^ This difference was explained by hypothesizing a role for eukaryotic frataxin as an allosteric modulator that stimulates PLP‐based chemistry and sulfur delivery to the scaffold protein for the cluster synthesis.^20^ While the *in vitro* work sets robust foundations toward a structural understanding of the frataxin function, it has been difficult to validate these findings *in vivo* or at least in a more complex environment that may contain all the relevant components.

To understand the prokaryotic versus eukaryotic differences, genetic studies have been designed by engineering strains that allowed depletion of individual genes from the *isc* operon of *E. coli*: a CyaY‐dependent strain was created and used to investigate the function of several components of the Fe–S cluster biogenesis machine *in vivo*.^21^ However, although elegant, this study works on timelines very different from those observed *in vitro* and has the limitation of not permitting supplementation of specific components within the same experiment.^21^, ^22^, ^23^ The use of novel approaches that could capture the complexity of the *in vivo* system, allowing at the same time an *in vitro* kinetics analysis of the desulfurase, is thus strongly required to clarify the regulatory mechanism of Fe–S cluster biogenesis machine in different organisms.

For this purpose, we describe, here, the use of a metabolomic approach based on following the iron–sulfur cluster formation kinetics directly in cellular lysates. This *ex vivo* approach has the advantage of retaining all the complexities of the *in vivo* system while being closer to the highly controlled *in vitro* situation. It also allows not only ad hoc depletion of selective proteins taking advantage of previously engineered mutants, but also a posteriori supplementation of specific components providing a higher level of control and flexibility. In the present study, we used a well‐known colorimetric assay that permits to follow the formation of sulfide (the by‐product of the IscS desulfurase activity), thus probing the desulfurase activity of the enzyme. This choice reports only indirectly on the formation of the iron–sulfur clusters but permits to focus on the specific desulfurase role which ultimately leads to the formation of the persulfide. Our approach allowed us to compare the effect of frataxin depletion in *ex vivo* prokaryotic cells, using *E. coli* as a model system. We demonstrate that the co‐presence of bacterial frataxin and iron acts as the effectors of cluster formation and confirms that prokaryotic CyaY is an inhibitor of IscS activity.

## 2 EXPERIMENTAL PROCEDURES

### 2.1 Bacterial strains and growth conditions

The *E. coli* parental strain (DV901) and its Δ*cyaY* (DV925) derivative have been previously described.^22^, ^24^ The Δ*iscS* (BP547) *E. coli* mutant strain was obtained by introducing the Δ*iscS*::kan KEIO mutation into DV901 by P1 transduction and confirmed by PCR. The lack of IscS in BP547 was also confirmed immunologically by Western blot. *E. coli* strains were grown in Luria‐Bertani (LB)‐rich medium at 37°C under aerobiosis. When required, kanamycin and ampicillin were used at 25 and 50 μg/ml, respectively.

### 2.2 Protein purification

*E. coli* CyaY and IscS were purified as previously described.^5^, ^25^, ^26^ In short, they were expressed in *E. coli* as His‐tagged or His‐tagged‐glutathione‐S‐transferase fusion proteins and purified by affinity chromatography using Ni‐NTA agarose gel (Qiagen, Milan, Italy). All purification steps were carried out in the presence of 20‐mM β‐mercaptoethanol. The collected proteins were cleaved overnight from their tags by Tobacco Etch Virus protease and further purified by gel‐filtration chromatography on a Superdex 75 10/60 column (GE Healthcare, Milan, Italy). Protein purity was checked by SDS‐polyacrylamide gel electrophoresis and mass spectrometry.

### 2.3 Cell lysate preparation

*E. coli* wild‐type (DV901), *ΔcyaY* (DV925), and *ΔiscS* (BP547) strains' inoculum was prepared in Luria Broth medium to an OD_600_ of 0.7. The cells were harvested and resuspended in a lysis buffer (20 mM Tris–HCl pH 8, 150 mM NaCl, 3 mM DTT, 0.2% IGEPAL) containing DNAse I and lysozyme. The cell extracts were obtained by centrifuging at 16,000 *g for 20 min at 4°C after sonication. The extracts were used immediately after preparation to avoid any possible protein denaturation. The total amount of proteins was quantified in *E. coli* extracts by Bradford assays.^27^

### 2.4 Western blots for estimate of the IscS expression levels

Western blots were performed to detect and quantify IscS. Aliquots (25 μg) of total proteins in the various cell extracts were separated by SDS‐PAGE by using Mini‐Protean TGX gels (Bio‐Rad, Milan, Italy) and electrotransferred to polyvinylidene difluoride (PVDF) membranes (Trans‐Blot Turbo Transfer Pack, Bio‐Rad, Milan, Italy). The membranes were incubated with a rabbit anti‐IscS polyclonal antibody kindly provided by Prof. Silke Leimkühler (University of Potsdam, Germany), and with a commercial rabbit anti‐GroES polyclonal antibody (Abcam, Cambridge, UK), prior to their incubation with goat anti‐rabbit HRP‐conjugated secondary antibody (Abcam, Cambridge, UK). The housekeeping protein GroES was used as a marker of efficient cell lysis and of the IscS expression level. IscS and GroES were detected by chemiluminescence using the Clarity Western ECL substrate (Bio‐Rad, Milan, Italy), followed by their densitometric quantification using the image analysis software Bio‐Rad Image Lab Software 6.1.0. The Western blots were performed in triplicates.

### 2.5 Kinetics experiments

Kinetics experiments were performed in triplicates using either purified proteins or clear cell extracts in 20 mM Tris–HCl buffer at pH 8.0, in the presence of 150 mM NaCl and 3 mM DTT. Reaction mixtures of 2.5 ml contained, according to the specific assay, either 500 nM IscS or, in the case of extracts, 10 mg of total proteins. Cysteine (1 mM) was added to start the reaction. Aliquots of 0.25 ml were collected for analysis at different time points. Supplementation experiments in which Fe^3+^ (in the form of ferric chloride) and/or CyaY were added to the lysates (1 mM and 10 μM, respectively) were carried out. Additional experiments carried out with 25 μM and 200 μM Fe^3+^ were also attempted, but they resulted in a signal‐to‐noise ratio too poor to be taken into account, presumably because of the iron (III) low solubility. Control experiments were performed in the presence/absence of cysteine on previously boiled cell extracts.

### 2.6 Colorimetric assay

Quantification of sulfide formation was carried out according to the guidelines defined by literature standards^28^, ^29^, ^30^ by the N,N‐dimethyl‐p‐phenylenediamine (DMPD) colorimetric assay, conveniently set up to minimize the dispersion of hydrogen sulfide. The test is based on the production of sulfide by IscS, through an oxidative coupling reaction using DMPD and ferric chloride dissolved in hydrochloric acid. The reaction exploits the oxidative potential of ferric chloride. The reaction product is methylene blue which, under oxidative environment, has an absorbance at 650–670 nm. The solution of DMPD, of a dark violet color, was carefully preserved from light and oxygen to prevent degradation. Aliquots of the various reaction mixtures were collected at different time points into 0.5‐ml tubes with sealed caps. The reaction was stopped by 70‐mM NaOH and 4‐mM ZnCl_2_. The negative control was prepared by stopping the reaction immediately after cysteine addition. Color development occurred after addition of DMPD (from a 20 mM stock solution in 7.2 M HCl) and FeCl_3_ (from a 30 mM stock solution in 1.2 M HCl), at a final concentration of 2.0 and 3.0 mM, respectively. After 20‐min incubation in the dark, samples were analyzed spectrophotometrically at 650 nm to detect the amount of methylene blue. The amount of sulfide produced by IscS was calculated by using a molar extinction coefficient for methylene blue at 650 nm of 19,000 M^−1^ cm^−1.^ ^28^

## 3 RESULTS

### 3.1 Tuning the methodology: Measurement of purified IscS kinetics *in vitro*

**FIGURE 1:**
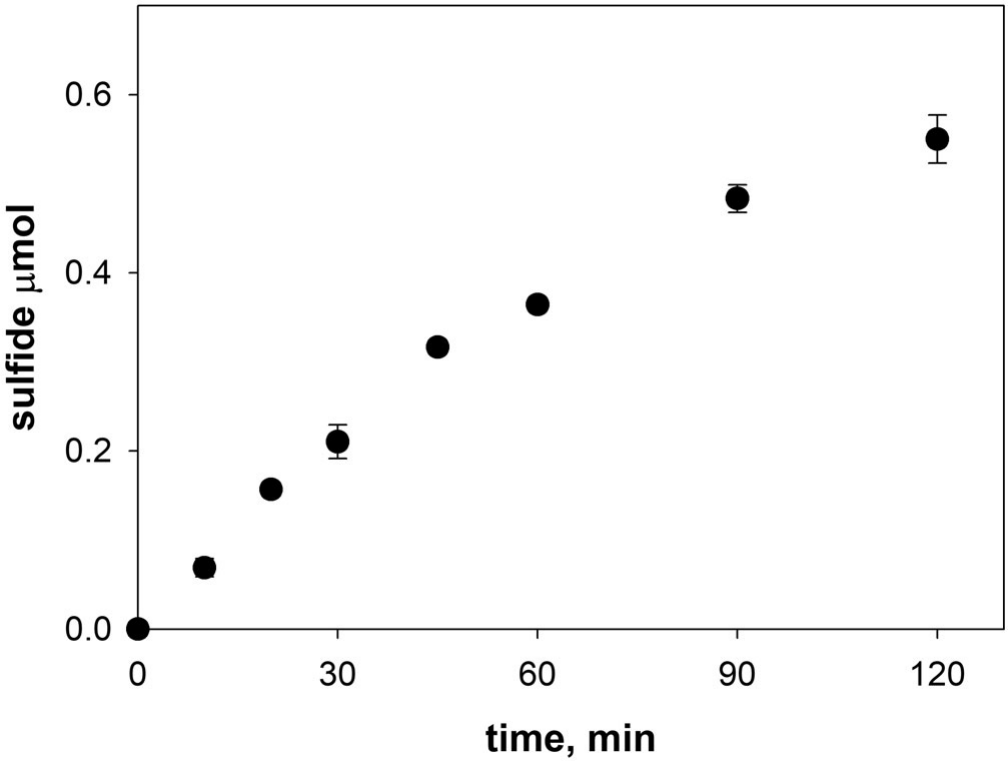
Purified IscS desulfurase *in vitro* kinetics. IscS activity was measured by a colorimetric assay based on the reaction between hydrogen sulfide and N,N‐dimethyl‐p‐phenylenediamine in the presence of iron (III) to form methylene blue that allows the spectroscopical quantification at 650 nm of the sulfide produced by IscS desulfurase. The amount of sulfide produced by IscS was calculated by using a molar extinction coefficient for methylene blue at 650 nm of 19,000 M^−1^ cm^−1^

The well‐established methylene blue assay was used to assess sulfide formation.^28^, ^30^ Initially, we calibrated the methodology using the purified desulfurase IscS. The reaction was started by adding the substrate (cysteine) to IscS in 20 mM Tris–HCl buffer, pH 8.0, in the presence of 150 mM NaCl and 3 mM DTT. Aliquots were collected at different time points and analyzed. Sulfide formation was detected as a progressive increase of absorbance values at 650 nm and by the appearance of the blue color as expected by the method (Figure 1). The plateau was reached ca. 120 min after the addition of the substrate. These results are fully consistent with those carried out following the reaction by circular dichroism or UV spectroscopy which measure the formation of the iron–sulfur cluster.^4^, ^24^ This important result told us that we can confidently use the colorimetric assay quantitatively and, more importantly, that the reaction rate‐limiting step of the reaction is sulfide formation.

### 3.2 Purified IscS activity *in vitro* is independent from iron

**FIGURE 2:**
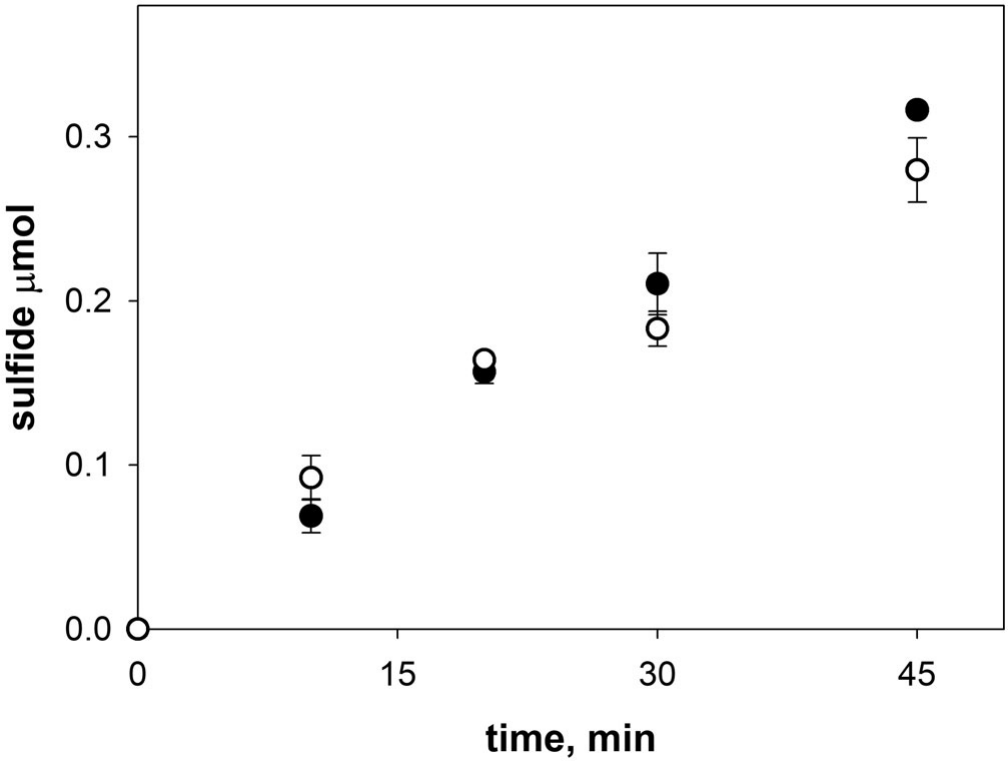
Effect of iron on the IscS enzymatic activity. Purified IscS *in vitro* kinetics was assessed by measuring the kinetics of sulfide formation in the presence (white circles) and in the absence (black circles)

The effect of iron on the IscS enzymatic activity was then assessed *in vitro* by adding a large excess of iron (1 mM FeCl_3_) to the reaction mixture. Aliquots at different time points were collected and analyzed. The comparison of IscS kinetics in the absence and in the presence of iron showed minimal differences (Figure 2), suggesting that iron alone does not affect nor does it alter IscS activity. IscS kinetics in the presence of iron was measured only over a limited time range (45 min). This was because the progressive accumulation over time of the black insoluble iron sulfide salts strongly interferes with the colorimetric assay at longer reaction times. The results obtained were highly reproducible, thus generating a solid prerequisite for attempting the measurement of IscS activity in bacterial lysates.

### 3.3 IscS desulfurase activity in *E. coli* cell lysate

**FIGURE 3:**
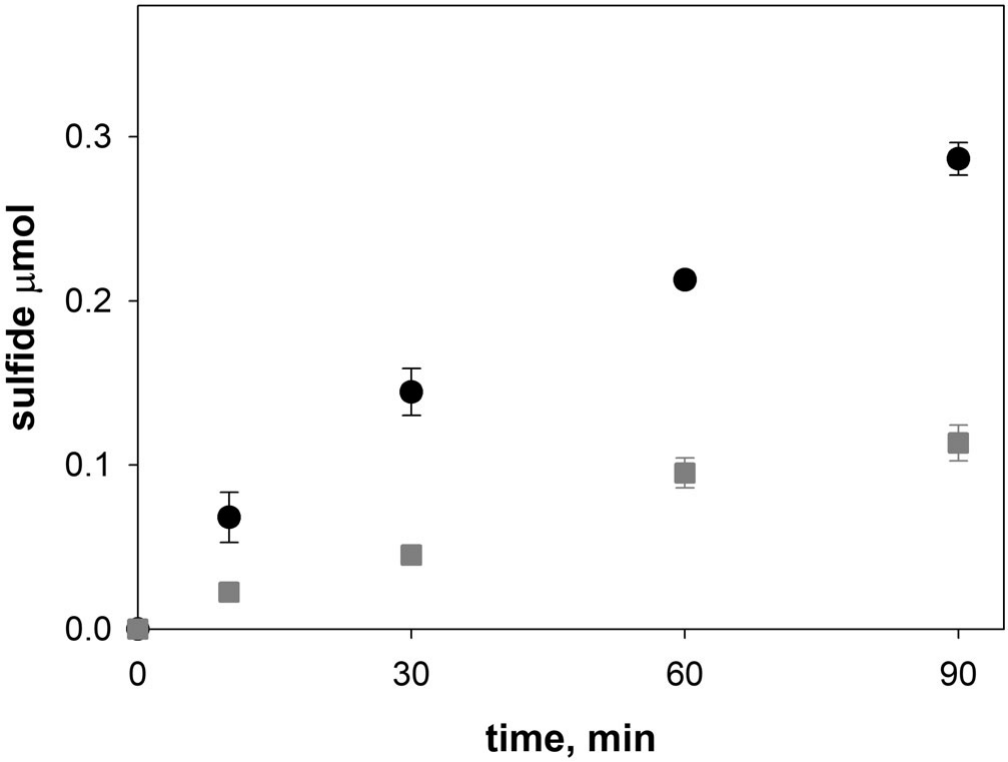
Investigation of IscS desulfurase kinetics in bacterial cell lysates. Sulfide formation was assessed over time in cell extracts from *E. coli* wild‐type (DV901; black circles) and ΔIscS (BP547; gray squares) strains

The protocol used to measure IscS activity *in vitro* was subsequently applied on bacterial cell lysates to investigate IscS kinetics within the complexity of an *in vivo* environment. It is noteworthy to consider that IscS is not the only enzyme in the cell able to transfer a thiol group from cysteine and release alanine as a by‐product. Various other enzymes such as SufS and CsdA have been widely characterized and proven to be able to remove the thiol group from cysteine in *E. coli*.^31^ It was, therefore, necessary to define the contribution over IscS of these desulfurases to sulfide production in cell extracts. For this purpose, sulfide production was measured and compared in cell extracts both from *E. coli* wild‐type (DV901) and *ΔiscS* (BP547) strains. The latter presents a deletion of the gene that encodes IscS.^32^ The two bacterial strains were grown individually but simultaneously using the same medium and were harvested at the same optical density. The mutant strain presented small colonies and much slower growth, highlighting a high level of stress. IscS kinetics measurements in the two *E. coli* strains were carried out using the same amount of total proteins (10 mg) as quantified by the Bradford colorimetric assay. Different aliquots were collected and analyzed at various time points. A much lower sulfide formation was observed for the *ΔiscS* cell extract when compared to the wild‐type strain, which suggests that IscS is the main source of desulfuration and that *E. coli* needs relatively little desulfurase activity to be viable (Figure 3). This is an important result per se. IscS deletion within the mutated strain (ΔIscS) was independently confirmed immunologically by Western blot as previously described^32^ (Figure S1). We, thus, concluded that the large difference in sulfide production between the wild‐type and ΔIscS *E. coli* strains tells us that the majority of sulfide produced in the cell is the consequence of the IscS activity, with a lower contribution from other desulfurases. We can, thus, be confident that any further study to investigate the activity of IscS in cell lysates can be ascribed directly to the effect of the *isc* operon components.

### 3.4 Does CyaY affect the IscS activity in *E. coli* wild‐type cell extracts?

**FIGURE 4:**
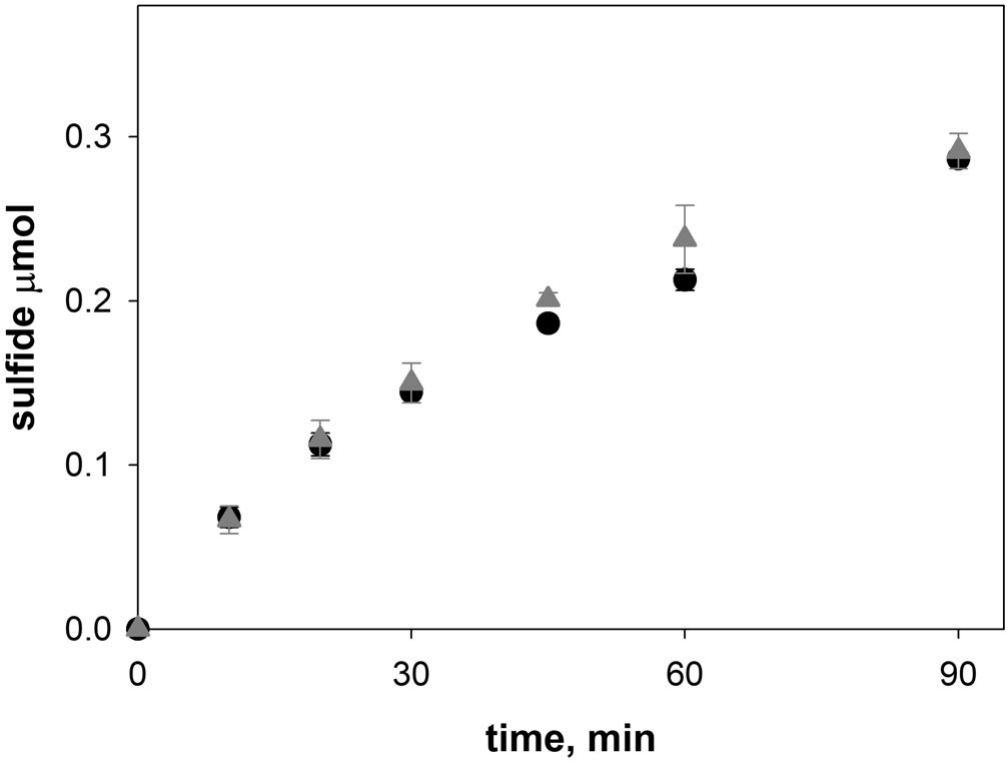
Effect of CyaY on the IscS desulfurase activity in cell extracts. Kinetics of sulfide formation was followed spectrophotometrically at 650 nm in *E. coli* wild‐type (DV901 strain, black circles) and ΔCyaY (DV925 strain; gray triangles) cell extracts. The amount of sulfide was calculated by using a molar extinction coefficient for methylene blue at 650 nm of 19,000 M^−1^ cm^−1^

IscS activity was then measured in cell extracts from *E. coli* comparing the behavior of wild‐type (DV901) and a strain lacking CyaY (*ΔcyaY* (DV925) mutant). The IscS expression levels in the wild‐type and in the ΔCyaY strains were assessed immunologically as previously described^21^ (Figure S2) and resulted comparable. Kinetics of sulfide formation showed no significant differences in IscS activity in both strains (Figure 4). This is a clear indication that IscS is active in the extract also in the absence of CyaY and that CyaY does not work as a regulator under these conditions. These results could be surprising considering that CyaY has been shown to inhibit IscS activity in *in vitro* experiments.^33^ The discrepancy could be explained considering that the *in vitro* experiments were by necessity carried out in the presence of only a few components of the Fe–S cluster machine. In the lysates, it is reasonable that other components could be able to compensate for the absence of CyaY.

### 3.5 Role of iron in the IscS activity in wild‐type and *ΔcyaY E. coli*

**FIGURE 5:**
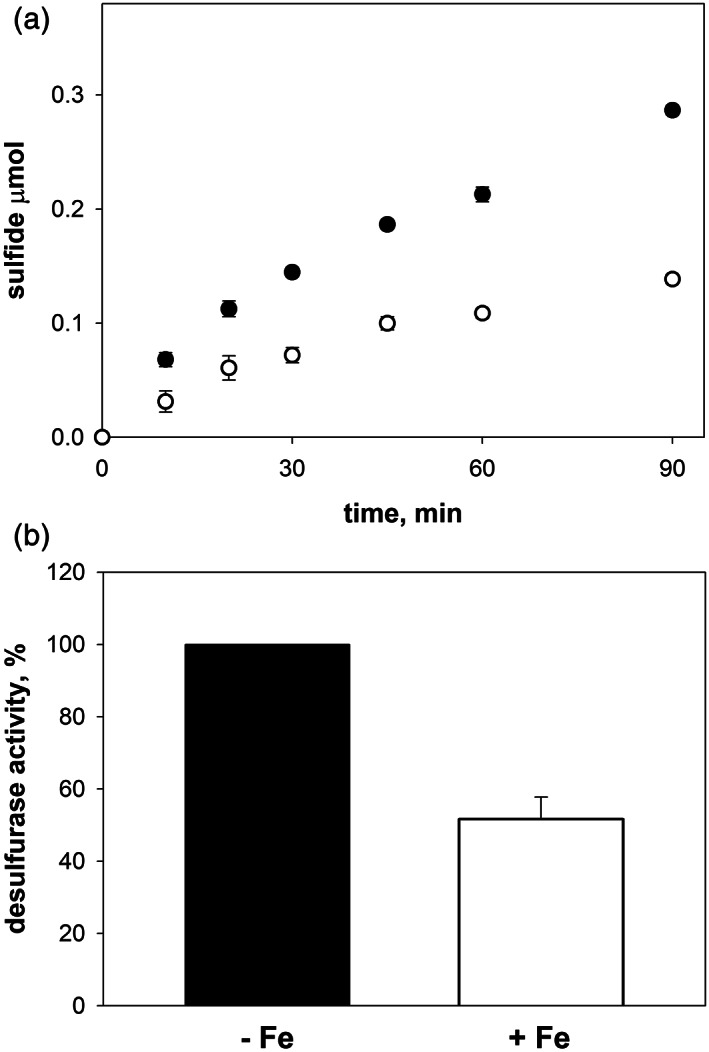
Kinetics of sulfide formation in cell extracts from the *E. coli* wild‐type strain. (a) The kinetics was followed by spectrophotometric measurements of methylene blue in the absence (black circles) and in the presence (white circles) of iron. (b) Comparison of the initial rates of the desulfurase reaction in the *E. coli* wild‐type cell lysate in the absence (black bar) and in the presence (white bar) of iron

The similarity of the kinetics observed in the cell extracts of the *E. coli* wild‐type (DV901) and the *ΔcyaY* (DV925) strains required further investigation. Since iron has been suggested as a potential effector in prokaryotes of the Fe–S cluster biogenesis,^5^, ^6^, ^7^, ^18^ the sulfide formation kinetics in cell extracts was investigated in the presence and in the absence of exogenously added iron. The reaction was started by adding the substrate to the cell extracts. The enzymatic activity of IscS was initially measured in the cell lysate of the *E. coli* wild‐type (DV901) strain in the absence and in the presence of exogenously added iron (Figure 5). The results showed a significant decrease in the initial rates upon addition of an iron excess to the *E. coli* wild‐type cell extracts when compared to the control where no exogenous iron was added. Since it is also worth bearing in mind that the lysates contain anyway physiological concentrations of iron, these data suggest an inhibitory effect of an iron excess on the IscS desulfurase activity which, under our conditions, corresponds to a decrease of the initial rates of around 50%.

**FIGURE 6:**
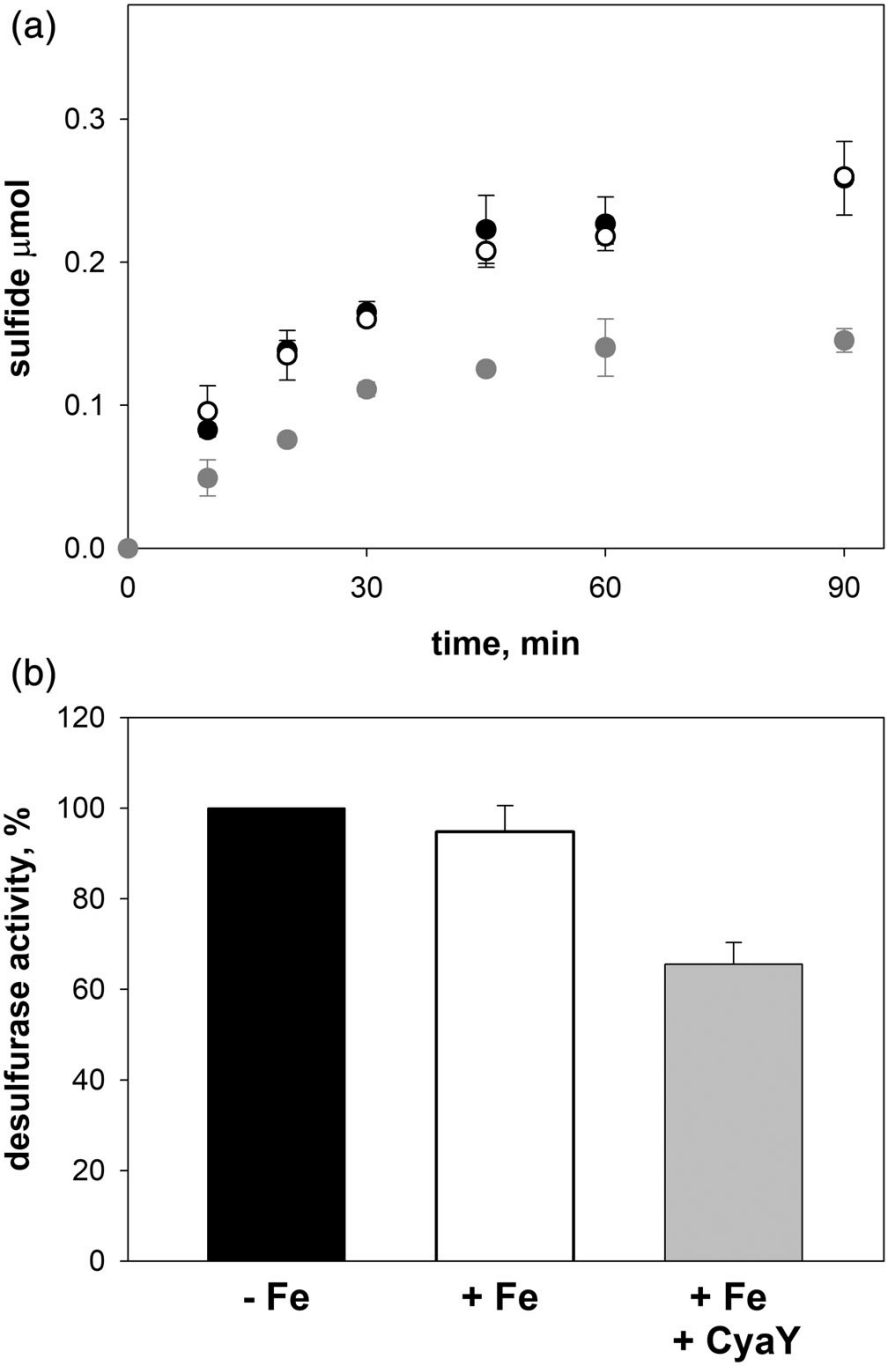
Effect of the co‐presence of CyaY and iron on the kinetics of sulfide formation in cell extracts from the *E. coli* ΔCyaY strain. The concentrations of added CyaY and iron (as ferric chloride) were 10 μM and 1 mM, respectively. (a) The kinetics of sulfide formation was followed by spectrophotometric measurements of methylene blue at 650 nm in the absence of iron (black circles), in the presence of iron (white circles), and in the simultaneous presence of CyaY and iron (gray circles). (b) Comparison of the initial rates of the desulfurase reaction in the *E. coli* ΔCyaY cell lysate in the absence of iron (black bar), in the presence of iron (white bar), and in the presence of purified CyaY and iron (gray bar)

The kinetics of sulfide formation was then measured in lysates of the *ΔcyaY* (DV925) deletion mutant in the presence and in the absence of iron. This kinetics showed identical initial rates (Figure 6), thus indicating that iron requires the co‐presence of CyaY to act as an effector of the enzymatic activity of IscS and vice versa. To prove this hypothesis, we carried out kinetics measurements after adding simultaneously purified CyaY and iron to the extracts of *E. coli ΔcyaY* cells (DV925). The amount of CyaY added to the reaction mixture was fixed a priori based on literature data.^15^, ^26^ Sulfide quantification at various time points clearly showed that the simultaneous addition of CyaY and iron significantly affects the initial rate of IscS activity with respect to the kinetics of IscS in the presence and in the absence of iron (Figure 6). The initial rates of the IscS desulfurase activity in the ΔCyaY cell lysates in the absence of exogenous iron, in the presence of exogenous iron, and in the presence of both exogenous iron and CyaY show an inhibition of 40% of IscS activity when purified CyaY and iron were added together to the ΔCyaY (DV925) cell lysate.

These results conclusively demonstrate that the CyaY action is iron modulated.

## 4 DISCUSSION

Our understanding of the mechanism of iron–sulfur cluster formation has been slowed down by the ambivalence between *in vitro* and *in vivo* studies which have often provided apparently contradicting results. In this work, we present a novel approach to dissect the enzymatic formation of sulfide that is at the midpoint between *in vitro* and *in vivo* experiments. This is based on using carefully designed genetic tools which allow the selective depletion of specific genes, in our case in a bacterial operon, and the establishment of the effects by following enzymatic kinetics.^21^ We believe that the ability to measure the activity of IscS/Nfs1 under conditions as close as possible to the in‐cell environment, but yet sufficiently controlled, may allow a more complete understanding of the correct interplay between the individual components of the Fe–S cluster biogenesis machine both in prokaryotes and eukaryotes.

We adopted here a colorimetric assay because this is simple, inexpensive, and well consolidated by several different groups. Alternative detection approaches are nevertheless possible, as, for instance, it could be the use of metabolomic mass spectrometry to follow the formation of alanine. This approach could reliably be used to follow alanine formation from the reaction catalyzed by IscS/NFS1 in models where the genes encoding proteins from this pathway have been depleted by exploiting ^13^C labeled cysteine as the substrate, to be able to distinguish the iron–sulfur cluster pathway from others. We already carried out preliminary attempts along these lines that fully established the feasibility of this method (Pastore and Adinolfi, unpublished results). What is important anyway is that, independently of the detection methodology selected, this method provides a new powerful tool that is in between the minimalistic *in vitro* approach and the full complexity of *in vivo* studies. The *ex vivo* strategy also offers the possibility to supplement specific components to understand their contribution to the system, as we have seen in the experiments in which we compared the effects of the absence and the added presence of CyaY.

Which conclusions can we draw from this study? We have observed that the desulfurase activity of *E. coli* is strongly dominated by IscS, making the contribution of other desulfurases secondary. This is per se an important observation. We have also shown that the bacterial desulfurase IscS activity is independent from the presence of CyaY as already extensively proven in *in vitro* studies.^6^ The IscS activity is also independent from the presence of iron. The presence of CyaY does not per se affect the enzymatic activity, as seen by comparing the kinetics in the wild‐type strain and in the ΔCyaY mutant. Conversely, an inhibitory effect becomes clear when an excess of iron is added to the wild‐type strain which does of course contain CyaY and physiologic iron contents. This finding was conclusively supported by the complementation experiment in which both iron and CyaY were endogenously added to the lysates. A regulatory role of Fe^2+^ has been suggested before by *in vitro* studies using purified proteins,^5^, ^7^ but to the best of our knowledge, there is no information on the ability of iron to regulate directly IscS kinetics *in vivo*. While our results reproduce *in vitro* evidence, they also tell us that no other components intervene to modulate the regulation and that the allosteric regulation mediated by the presence of CyaY needs the presence of iron. A similar behavior was observed when the reconstitution of the Fe–S cluster on IscU was studied at a different iron concentration.^5^, ^7^ Thus, the inhibitory effect of CyaY on cluster formation is fully supported also by our *ex vivo* data.

We believe that the same approach can, in principle, be successfully applied in the future also to eukaryotic cell lysates leading to a possible significant advancement in clarifying the frataxin function. Thus, in conclusion, we do hope that the metabolomic approach reported here will contribute to develop more complex holistic studies that will provide new information useful for defining the role of frataxin in the cell that is an essential step to identify a possible cure for this deadly disease.

## Supporting information


**Figure S1** Western blot demonstrating the absence of IscS within the mutated strain *ΔiscS* (BP547). The blot shows IscS (solid line) and groES (dashed line) in cell lysates from *E. coli* wild‐type BP230 strain (lane 3) and *ΔiscS* (BP547) mutant strain (lane 4). Lane 1 reports molecular weight markers, while lane 2 reports purified IscS. Proteins in the various samples were separated by SDS‐PAGE and electrotransferred onto a PVDF membrane. The membrane was cut into two parts that were used for the immunodetection of either IscS or groES.
**Figure S2** IscS quantification in wild‐type and CyaY‐deleted strain *ΔcyaY* (DV925). The Western blot shows IscS (solid line frame) and GroES (dashed line frame) in cell lysates from *E. coli* wild‐type (DV901) strain (lane 2) and *ΔcyaY* (DV925) mutant strain (lane 3). Lane 1 reports pre‐stained molecular weight markers. Proteins in the various samples were separated by SDS‐PAGE and electrotransferred onto a PVDF membrane. The membrane was cut into two parts that were used for the immunodetection of either IscS or groES. Densitometric image analysis by the Bio‐Rad Image Lab Software allowed assessing the intensity of the various protein bands and calculation of the GroES to IscS ratio in both strains. The GroES to IscS ratio in *ΔcyaY* (DV925) cell lysate is 1.91 ± 0.09 A.U, while GroES to IscS ratio in wild‐type cell lysate is 1.71 ± 0.08 A.U.
