# Supplementary material for: Towards a metabolomic approach to investigate iron–sulfur cluster biogenesis
Source: IUBMB Life. 2022 Apr 27;74(7):715–22. doi: 10.1002/iub.2618 (PMC9325406; doi:10.1002/iub.2618)
Supplement: Supplementary file 1 — Figure S1 Western blot demonstrating the absence of IscS within the mutated strain ΔiscS (BP547). The blot shows IscS (solid line) and groES (dashed line) in cell lysates from E. coli wild‐type BP230 strain (lane 3) and ΔiscS (BP547) mutant strain (lane 4). Lane 1 reports molecular weight markers, while lane 2 reports purified IscS. Proteins in the various samples were separated by SDS‐PAGE and electrotransferred onto a PVDF membrane. The membrane was cut into two parts that were used for the immunodetection of either IscS or groES. Figure S2 IscS quantification in wild‐type and CyaY‐deleted strain ΔcyaY (DV925). The Western blot shows IscS (solid line frame) and GroES (dashed line frame) in cell lysates from E. coli wild‐type (DV901) strain (lane 2) and ΔcyaY (DV925) mutant strain (lane 3). Lane 1 reports pre‐stained molecular weight markers. Proteins in the various samples were separated by SDS‐PAGE and electrotransferred onto a PVDF membrane. The membrane was cut into two parts that were used for the immunodetection of either IscS or groES. Densitometric image analysis by the Bio‐Rad Image Lab Software allowed assessing the intensity of the various protein bands and calculation of the GroES to IscS ratio in both strains. The GroES to IscS ratio in ΔcyaY (DV925) cell lysate is 1.91 ± 0.09 A.U, while GroES to IscS ratio in wild‐type cell lysate is 1.71 ± 0.08 A.U. [file IUB-74-715-s001.docx]

**Supplementary Information**

**
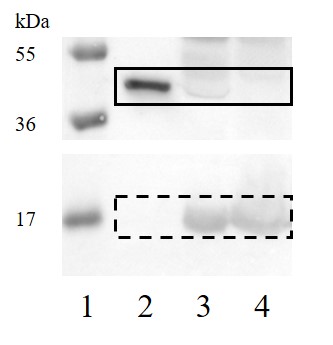
**

**Figure S1 –** Western blot demonstrating the absence of IscS within the mutated strain *ΔiscS* (BP547). The blot shows IscS (solid line) and groES (dashed line) in cell lysates from *E. coli* wild-type BP230 strain (lane 3) and *ΔiscS* (BP547) mutant strain (lane 4). Lane 1 reports molecular weight markers while lane 2 reports purified IscS. Proteins in the various samples were separated by SDS-PAGE, and electrotransferred onto a PVDF membrane. The membrane was cut into 2 parts that were used for the immunodetection of either IscS or groES.

**
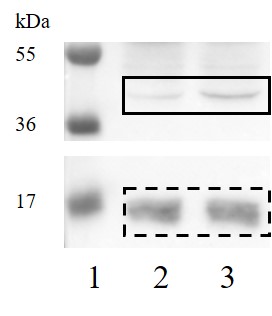
**

**Figure S2 –** IscS quantification in wild type and CyaY deleted strain *ΔcyaY* (DV925). The Western blot shows IscS (solid line frame) and GroES (dashed line frame) in cell lysates from *E. coli* wild-type (DV901) strain (lane 2) and *ΔcyaY* (DV925) mutant strain (lane 3). Lane 1 reports pre-stained molecular weight markers. Proteins in the various samples were separated by SDS-PAGE, and electrotransferred onto a PVDF membrane. The membrane was cut into 2 parts that were used for the immunodetection of either IscS or groES. Densitometric image analysis by the Bio-Rad Image Lab Software allowed assessing the intensity of the various protein bands and calculation of the GroES to IscS ratio in both strains. The GroES to IscS ratio in *ΔcyaY* (DV925) cell lysate is 1.91 ± 0.09 A.U. while GroES to IscS ratio in wild type cell lysate is 1.71 ± 0.08 A.U..
